# Effects of Combined Transcranial Direct Current Stimulation with Cognitive Training in Girls with Rett Syndrome

**DOI:** 10.3390/brainsci10050276

**Published:** 2020-05-02

**Authors:** Rosa Angela Fabio, Antonio Gangemi, Martina Semino, Aglaia Vignoli, Alberto Priori, Maria Paola Canevini, Gabriella Di Rosa, Tindara Caprì

**Affiliations:** 1 Department of Clinical and Experimental Medicine, University of Messina, Via Bivona, 98100 Messina, Italy; rafabio@unime.it (R.A.F.); antgangemi@unime.it (A.G.); 2 Centro AIRETT Ricerca e Innovazione (CARI), Research and Innovation Airett Center, 37100 Verona, Italy; martina_semino@hotmail.it; 3 Department of Health Sciences, Child Neuropsychiatry Unit, Epilepsy Center, San Paolo Hospital, Universitàdegli Studi di Milano, 20142 Milan, Italy; aglaia.vignoli@ao-sanpaolo.it (A.V.); maria.canevini@asst-santipaolocarlo.it (M.P.C.); 4 Aldo Ravelli Research Center for Neurotechnology and Experimental Neurotheraputics, Department of Health Sciences, University of Milan, 20142 Milan, Italy; alberto.priori@unimi.it; 5 Division of Child Neurology and Psychiatry, G. Martino Hospital, University of Messina, Via Consolare Pompea, 46046 Messina, Italy; gdirosa@unime.it

**Keywords:** Rett syndrome, tDCS, attention, language, cognitive training

## Abstract

Background: Transcranial Direct Current Stimulation (tDCS) combined with traditional rehabilitative techniques has not been widely applied to Rett Syndrome (RTT). The aim of this study was to examine the effects of combined cognitive traditional training with tDCS applied to attention and language measures in subjects with RTT. Methods: 31 subjects with RTT were randomly allocated into two groups: non-sham tDCS (*n* = 18) and sham tDCS (*n* = 13). The former received the integrated intervention non-sham tDCS plus cognitive empowerment during the treatment phase. The latter received sham stimulation plus cognitive empowerment. All participants underwent neurological and cognitive assessment to evaluate attention and language measures: before integrated treatment (pre-test phase), at the conclusion of the treatment (post-test phase), and at 1 month after the conclusion of the treatment (follow-up phase). Results: the results indicated longer attention time in the non-sham tDCS group compared to the sham tDCS group with a stable trend also in the follow-up phase; an increase of the number of vowel/phoneme sounds in the non-sham tDCS group; and an improvement in the neurophysiological parameters in the non-sham tDCS group. Conclusions: This study supports the use of tDCS as a promising and alternative approach in the RTT rehabilitation field.

## 1. Introduction

Rett syndrome (RTT) is a complex genetic disorder, caused by mutations in the X-linked gene encoding for a regulator of epigenetic gene expression, methyl CpG binding protein (MeCP2) [1,2]. Although mutations in MECP2 gene are the main cause of RTT disease, other mutations are associated with RTT, such as: FOXG-1 and CDLK-5 [3,4]. RTT is a rare disease and occurs almost exclusively in girls, affecting 1:10,000 people.

The clinical picture associated with typical RTT is defined by loss of hand use and language, with the development of gait abnormalities and hand stereotypies [5]. Although the loss of purposeful hand movements and speech, and regression of acquired cognitive and motor skills are the core symptoms associated with this syndrome, RTT phenotype includes several manifestations of associated comorbidity that affect different systems, for example: nervous, muscolo-skeletal, and gastro-enteric systems [6,7,8,9,10,11,12]. Given the co-presence of different disabilities, management of RTT symptoms and health-related problems require an expert team of multi-specialists to ensure good patient quality of life [13,14,15,16,17].

New promising therapeutic approaches have recently been developed in the rehabilitation of multiple disabilities through the use of non-invasive brain stimulation, such as transcranial direct current stimulation (tDCS). tDCS is a neurostimulation technique that modulates the resting threshold of neurons. tDCS involves delivering prolonged (10–20 min), low-intensity electric current (1–2 mA) directly through a pair of electrodes placed on the scalp. The electrical current produced by tDCS runs from one electrode to another electrode for the entire time that stimulation is delivered [18]. tDCS can operate as both an exciter or an inhibitor of the resting threshold of neurons. For excitation, it can make neurons more likely to charge if done in a manner to increase excitation by lowering their resting threshold. For inhibition, the resting threshold is raised, which makes neurons less likely to fire. Positive effects of tDCS have been observed in various disorders, for example: stroke, aphasia syndromes [19], and Alzheimer’s disease [20]. Several studies have indicated positive results of language training for post-stroke aphasia when combined with tDCS [21,22,23]. Promising results have also been obtained in the treatment of patients with cognitive and motor deficits, demonstrating that tDCS can enhance some aspects of cognition [24] and promote the recovery of motor deficits [25]. There is evidence supporting the use of tDCS when combined with traditional rehabilitative techniques in patients with neurological, motor, cognitive, and language disorders [26]. Due to these positive findings, the current methodological direction is to involve functional targeting in tDCS studies aimed at enhancing the effects of a particular training program by combining it with tDCS [27].

Although this combined approach is attracting considerable interest in the multiple disability area, due to positive results, it has not been widely applied to RTT [28,29].The work of Fabio, Gangemi, Caprì, Budden, and Falzone [29] is the first study designed to examine the neurophysiological and cognitive effects of tDCS in girls with RTT with chronic language impairments. The authors applied an integrated intervention: tDCS and cognitive empowerment applied to language to enhance speech production (new functional sounds and new words). Because maximal gains are usually achieved when tDCS is coupled with behavioural training, they applied tDCS stimulation on Broca’s area together with linguistic training. The results indicated a general enhancement in language abilities (an increase in the number of vowel/consonant sounds and words, and production and comprehension through discrimination), motor coordination (functional movements), and neurophysiological parameters (an increase in the frequency and power of alpha, beta and theta bands).

Overall, the initial studies on RTT only focused on establishing the role of integrated tDCS and cognitive empowerment on language skills, with positive findings. A possible explanation of these previous data is that the preliminary mechanism of tDCS is a subthreshold modulation of neuronal resting membrane potential, which induces a polarity-dependent modification of N-Methyl-daspartate (NMDA) receptor function [30] that plays a role in neuroplasticity [31]. Despite these encouraging results, other abilities, such as attention or memory, were not dealt with in depth. Hence, there is still a need for understanding how the tDCS, in combination with traditional training, can improve language and cognitive abilities in patients with RTT. This is crucial in supporting the use of tDCS as a promising alternative for rehabilitation in an RTT population. Currently, multiple therapies such as cognitive, physical, occupational, speech therapy, and others have been shown to be effective in promoting improved communication, motor control and cognitive abilities of subjects with RTT [32,33,34,35,36,37]. However, the question is open: What is missing is the knowledge about the exact combination of these trainings with tDCS and the extent of practice required to promote skill development. More in depth, it is important to define a protocol design that maximizes the results of both interventions. To advance the application of combined tDCS interventions also in RTT, this study has the goal of combining cognitive traditional training with tDCS applied to attention and language. The underlying logic of this study is that brain stimulations combined with traditional training can help neurological system functioning to maximize capacity, resulting in improved abilities over a long period. Precisely, we have examined if the tDCS could maximize treatment benefits in attention and language abilities. In accordance with previous studies [29], it was hypothesized that cognitive empowerment applied to language combined with tDCS would induce the production of new sounds and words. Also, it was hypothesized that the combined intervention would improve attention abilities, in terms of increased attention minutes.

## 2. Method

### 2.1. Participants

Thirty-five young girls and women with a diagnosis of RTT, ranging from age 13 to 35 years old (mean = 18.83 years, SD = 4.63), were recruited from the Italian Rett Association (AIRETT). Patients with FOXG1 and CDKL5 were excluded from the sample. Thirty-one participants were randomly allocated into two groups: non-sham tDCS (*n* = 18) and sham tDCS (*n* = 13). The former group received the integrated intervention non-sham tDCS plus cognitive empowerment during the treatment phase. While the latter tDCS group received sham stimulation plus cognitive empowerment.

All participants were born into a non-consanguineous marriage. An uncomplicated pregnancy and a full-term normal delivery were reported at the hospital. Regular immunizations were carried out. At birth, their weight and height were normal. At time of study, they were in a chronic phase with stable language, but with poor speech production and no changes after the third stage. They were not self-sufficient in walking or in everyday activities (eating, dressing and so on). All participants showed pervasive hand stereotypes. All attended schools or socio-educational centres. A general assessment was carried out by a psychologist through the Vineland Adaptive Behavior Scale (VABS) [38] and the Rett Assessment Rating Scales (RARS) [39]. Table 1 shows characteristics of two groups.

### 2.2. Study Design

This study employed a pre-test, post-test, follow-up design with two groups: non-sham tDCS group and sham tDCS group. In the pre-test phase, all participants underwent a neurological (EEG measure) and cognitive assessment to evaluate attention and language measures before the treatment (tDCS and cognitive empowerment). This same assessment was repeated once at the conclusion of the treatment (post-test phase) and once at 1 month after the conclusion of the treatment (follow-up phase). The scores obtained in the pre-test phase were compared with those observed in the post-treatment assessment phase and follow-up, to evaluate the effects of integrated intervention.

### 2.3. Assessment

The Vineland Adaptive Behavior Scales-Interview second edition (VABS) [38], the Rett Assessment Rating Scales (RARS) [39] and Fanzago’s test [40] were used.

VABS [38] is subdivided into four domains: communication; daily living; socialization; and motor skills. The interviewer asks general questions pertaining to the subject’s functioning in each subdomain and uses the responses to rate the examinee on each critical behaviour item (2: always present, 1: sometimes present, 0: seldom or never present). Typical interviews require approximately 1 h. A total score is computed by summing the individual ratings for each scale. The reliability of the scales was established as follows: split-half, 0.73–0.93 for the communication domain, 0.83–0.92 for daily living skills, 0.78–0.94 for socialization, 0.70–0.95 for motor skills, 0.84–0.98 for adaptive behaviour composite, and 0.77–0.88 for maladaptive behaviour (survey form) (0.80 and 0.90 for the Survey Form). The interrater reliability coefficients for the survey and expanded forms ranged from 0.62 to 0.75. Standard error of measurement ranged from 3.4 to 8.2 over the four domains, and from 2.2 to 4.9 for the Adaptive Behaviour Composite, on the survey form.

RARS [39] is a standardized scale used to evaluate subjects with RTT. It is organized into seven domains: cognitive, sensorial, motory, emotional, autonomy, and typical characteristics of the disease and of behaviour. The cognitive area consists of evaluations of attention, spatial orientation, temporal orientation, memory, eye contact, replying by smiling, shared attention, verbal and non-verbal communication; the sensorial area consists of eyesight and hearing; the motor area consists of position and movement of the body, movement of hands, scoliosis, problems in the feet; the emotional area refers to understanding emotions and the expression of emotions; the autonomy area refers to excretive control, feeding, ability to wash and dress; the typical characteristics of the disease and behaviour area refers to mood changes, convulsions, breathing problems, hyperactivity, anxiety, aggressiveness, bruxism, rolling of the eyes, epilepsy, aerophagia, muscular tension, feeding habits; and the overall impression area refers to the general evaluation of the symptoms of RTT (from no symptoms (1) to all the symptoms (4)). A total of 31 items was generated as representative of the profile of RTT. Each item is provided with a brief glossary explaining its meaning in a few words. Each item is rated on a 4-point scale, where 1 = within normal limits, 2 = infrequent or low abnormality, 3 = frequent or medium-high abnormality, and 4 = strong abnormality. Intermediate ratings are possible; for example, an answer between 2 and 3 points is rated as 2.5. For each item, the evaluator circles the number corresponding to the best description of the patient. After a patient has been rated on all 31 items, a total score is computed by summing the individual ratings. This total score allows the evaluator to identify the level of severity of RTT, conceptualized as a continuum ranging from mild symptoms to heavy deficits. Skewness and kurtosis values, calculated for the distribution of the total score, are 0.110 and 0.352, respectively. Distribution is found to be normal. Cronbach’s alpha is used to determine the internal consistency for the whole scale and subscales. Total alpha is 0.912, and the internal consistency of the subscales is high (from 0.811 to 0.934).

In these assessment phases, neurophysiological parameters are measured. EEG data are acquired using a gold-standard digital EEG amplifier (Cardinal Medical System) of 21 electrodes placed on the scalp of the participant according to the expected parameters of the international measuring system 10/20. Quantitative analysis is performed by tailor-made algorithms developed in Matlab code. The power spectral density (PSD) is evaluated by transforming the signal from the time domain to the frequency domain using the Welch method (Welch, 1967). A spectral analysis of EEG rhythms and the diffusion of the effect of the tDCS on all channels of registration are assessed.

### 2.4. Integrated Intervention: tDCS and Cognitive Empowerment

In this study, all participants received 20 min of tDCS plus cognitive empowerment for 10 daily sessions over a 1-week period. Precisely, at each treatment session, the 20 min of tDCS were applied concurrent with 20 min of cognitive empowerment. The electrodes were then removed.

In the non-sham group, participants received stimulation at 2 mA where a current flowed between the two electrodes. At 2 mA, it was possible that the current was excitatory, at both electrodes, as an effect of the parameters put in place. To localize the electrode placement, the 10:20 EEG system was used. The anode electrode (5 × 7 cm^2^) was placed horizontally over the C3 brain area (C3 of the 10–20 international EEG system). The cathode electrode (10 × 10 cm^2^) was positioned over the right supraorbital region. The electrodes were covered with rectangular saline-soaked sponge. We used a small Saline Solution Applicator Bottle (a 20 mL bottle) that allowed a slight control over the amount of liquid placed onto the sponges. Saline solution did not run down the scalp or spread over the participants’ hair. Once the anode electrode was placed over the target region (C3) it was secured using a rubber band. The cathode electrode was then secured in the same manner. The electrodes were then attached to a constant current stimulator (HDC type Company Stimulation Omicron T) using wires connected to corresponding anode/cathode ports.

In the sham tDCS Group, the current was ramped up and remained at 1 mA only for 30 s prior to ramping down. This sham tDCS did not affect the resting threshold of neurons, but ensured blinding of participants due to initial tingling sensations on the scalp. The sham tDCS administration procedure was the same as the non-sham tDCS. As stated above, sponges were soaked with saline solution and the electrodes were placed inside the sponge. Sponge-electrodes were then secured over the brain target previously identified, using a rubber band. Finally, the electrodes were energized using the corresponding anode and cathode wires connected to the stimulator.

With reference to cognitive empowerment, it was precise and well-structured procedures repeated every day for a long time. Treatment plans were based upon (1) level of impairment in behavior, cognition, and functional skills of patients, evaluated from a neuropsychological assessment, and (2) cognitive–behavioural strategies. During the treatment phase of this experiment, participants received 20 min of the combined intervention (tDCS plus cognitive empowerment) for 10 daily sessions over a 1-week period. The purpose of this combined intervention was to elicit the production of vowels, phonemes and words. With reference to cognitive empowerment, the used cognitive–behavioural strategies were: imitation procedures, prompting and generalization [29,37]. The stimuli consisted of coloured pictures of objects (food, animals, toys and familiar objects) with a dimension of 10 × 15 cm. In the production of vowels, phonemes and words, the participant was presented with an image. She was asked to look at it and give the matching word. Pictures of objects that started with vowels were first shown to her and subsequently those that started with a phoneme (b, c, d, m, p, t, s) and word (mummy, dad, cat, bee). If the participant spontaneously replied to the picture with the correct sound (or word), it was considered as a correct reply. If she spontaneously did not reply to the picture she was asked a simple question: “Look, what’s this? This is an ‘ape’ (Italian word for bee). It begins with “A”. Please repeat “A”. When the participant repeated the correct vowel, phoneme and/or a word three consecutive times, it was considered learned and was assigned the score of 1. After that, she was given the next stimulus. If she was not able to produce the target vocal sound three times, it was assigned the score of 0. The non-repeated sound was presented again the day after. The dependent variable was the number of correct answers.

During the administration of the cognitive–behavioural strategies, the attention time of the girl was examined. The time of attention started when the participant was sitting at a table and had the target on her right and a distractor on her left. She was asked to look at them and to try to produce the sounds (vowels, phonemes and words). When the participant stopped looking at the stimuli, or looked away around the environment, time of attention stopped being recorded (Fabio et al. 2018). In the integrated sham tDCS and cognitive empowerment intervention, the procedure was exactly the same as the non-sham tDCS and cognitive empowerment intervention.

## 3. Measures

### 3.1. Attention Measure

The total time spent by the girl looking at the stimuli presented was considered the parameter.

### 3.2. Production of Vowels, Consonants and Words with Elicited Denomination

The sum of the total vowels, phonemes and words was considered as the parameter. More precisely the scores were: If the vowel, consonant and/or word were produced correctly, the score was 1; if the word was partially produced, the score was 0.5; if the vowel, consonant and/or word was not produced, or produced differently, the score was 0.

### 3.3. Quantitative EEG Analysis

Quantitative analysis was performed by home-made algorithms developed in Matlab code. The power spectral density (PSD) was evaluated by transforming the signal from the time domain to the frequency domain using the Welch method [41]. PSDs were calculated for each epoch and were averaged. To begin with, the absolute total power of the signal and the absolute power of each band are worked out for each electrode. The considered bands were: theta (3.5–7 Hz), alpha (8–13 Hz) and beta (14–29 Hz).

### 3.4. Statistical Analysis

Data were analysed using SPSS Version 24.0 for Windows. Descriptive statistics of the dependent variables were tabulated and examined. Alpha level was set to 0.05 for all statistical tests. Bonferroni correction was applied for multiple comparisons. In the case of significant effects, the effect size of the test was reported. More precisely, for ANOVA, partial eta-squared (_p_η^2^) was used. The Greenhouse–Geisser adjustment for nonsphericity was applied to probability values for repeated measures.

With reference to the five parameters, time of attention, production of vowels, phonemes, words, and EEG parameters, seven repeated ANOVA measures were carried out with a between-subjects variable and a within-subjects variable: 2 (groups: non–sham tDCS vs sham tDCS) X 3 (phases: pre-test, post-test and follow-up).

## 4. Results

Table 2 shows descriptive statistics of attention, production of vowels, phonemes, words, and EEG parameters.

With reference to the time of attention, groups show no significant effect, F (1, 33) = 1.62; *p* < 0.08. A significant groups x phases interaction was found, F (2, 92) = 38.53; *p* < 0.0001, η2 = 0.12. This means that the time of general attention of participants increased after the intervention in the tDCS group, but remained stable in the sham tDCS group. Table 3 shows, more in detail, the post hoc comparisons, Bonferroni adjusted for each phase and parameter. As regards vowels, again no significant group effect was found, F (1, 33) = 1.91; *p* < 0.07, but there was a groups x phases interaction, F (2, 92) = 19.86; *p* < 0.0001, η2 = 0.12. This result indicates that the participants in the non-sham tDCS group produced a higher number of vowels than the sham group after the combined treatment and this enhancement remained stable also in the follow-up phase (see post-hoc comparison of Table 3).

Regarding the phoneme parameter, the results indicated the same trend. No significant group effect was found, F (1, 33) = 1.43; *p* < 0.11. A significant groups × phases interaction was found, F (2, 92) = 34.75; *p* < 0.0001, η2 = 0.11. This means that the non-sham tDCS produced a higher number of phonemes compared to the sham group. Post hoc comparison confirmed this result (Table 3). With reference to the number of words parameter, no statistically significant groups × phases interaction was found, but a positive trend was also present in this parameter.

**Figure 1:**
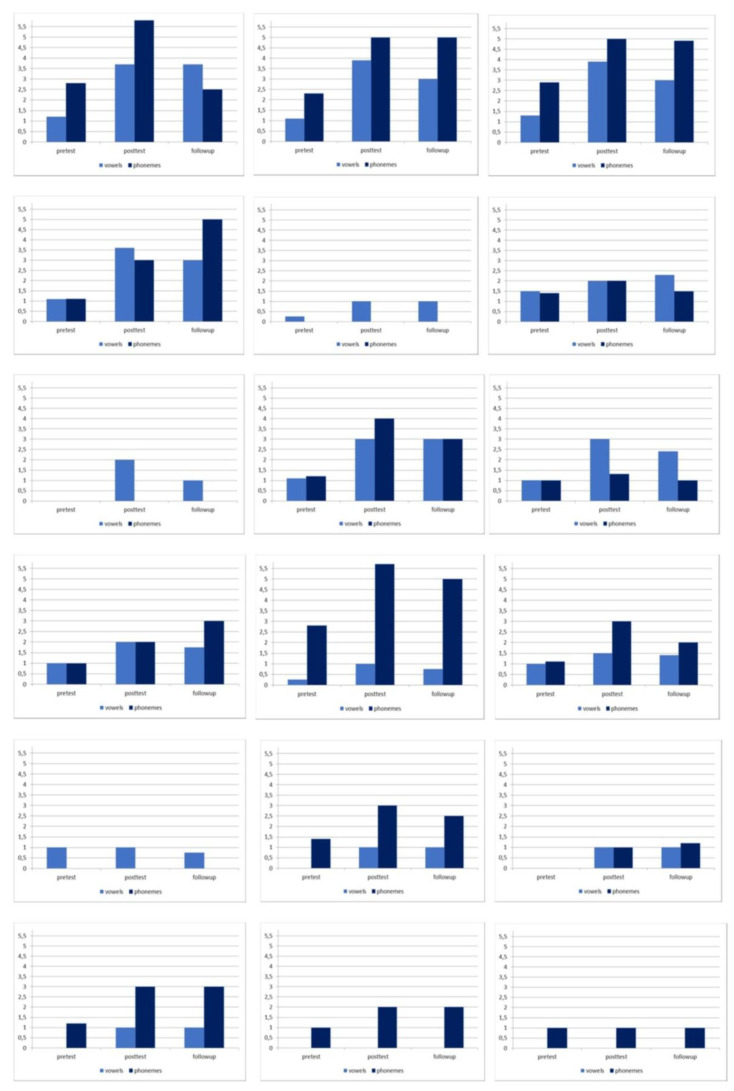
Visual representation of the individual’s change (from patient 1 to patient 18).

**Figure 2:**
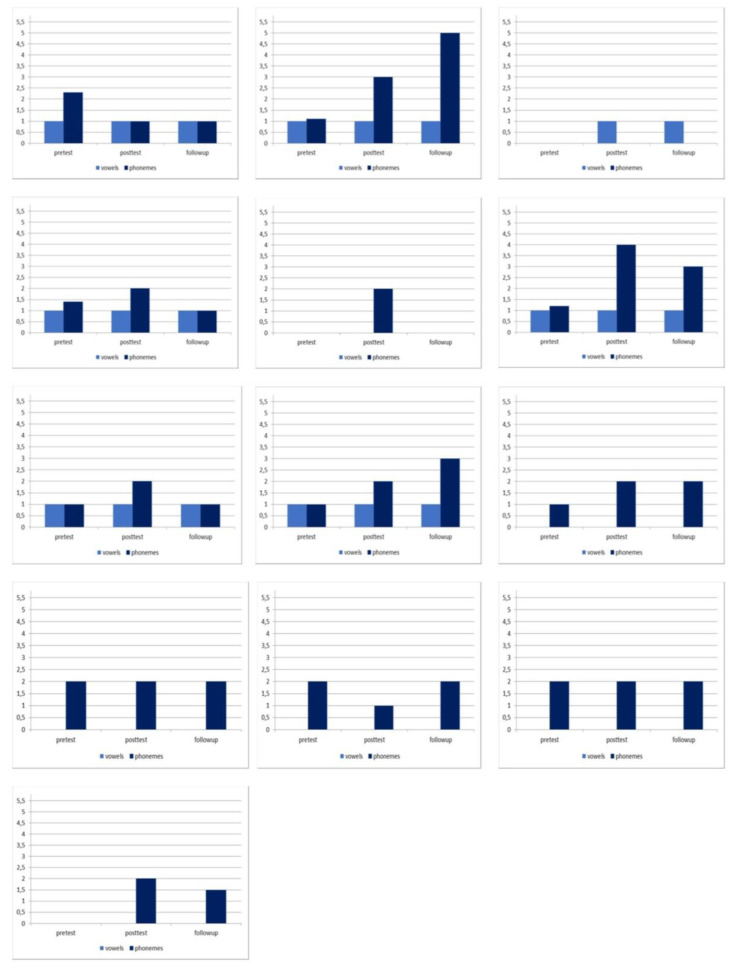
Visual representation of the individual’s change (from patient 19 to patient 31).

Figure 1 and Figure 2 show the visual representations of the individual’s change for each phase and vowels and phonemes parameters in the two groups.

Quantitative analysis of the neurophysiological parameter presented a significant rise in the frequency and power of alpha and beta bands. For the beta band, no group effect was found, F (1, 33) = 1.54; *p* < 0.08. There was a significant groups x phases interactions F (2, 92) = 19.86; *p* < 0.0001, η2 = 0.12. This means that the non-sham tDCS group showed an improvement in beta band. The same trend was found for the alpha band in the non-sham tDCS group; no group effect was found, F (1, 33) = 1.94; *p* < 0.08. There was a significant groups x phases interactions F (2, 92) = 35.86; *p* < 0.0001, η2 = 0.12. For theta bands, no group effect was found, F (1, 33) = 1.86; *p* < 0.08.

## 5. Discussion

The main aim of this study was to examine the effect of combined tDCS with cognitive training applied to attention and language abilities in subjects with RTT. As hypothesized, the present study demonstrates that the combined intervention improves both abilities. With reference to attention, it was found that the time of attention increased both after the non-sham tDCS intervention and after the sham tDCS intervention, but this increase is statistically significant in the non-sham tDCS group. This result is new in the RTT rehabilitation field, given that previous studies offered support for the use of tDCS in the improvement of language skills and the effects of combined cognitive training with tDCS have not yet been investigated for attention.

With reference to language, the results of the current work are consistent with previous studies [29], confirming the idea that speech training when combined with tDCS is more effective. Subjects in the non-sham tDCS group showed a higher number of vowels and phonemes than the sham group. Unexpectedly, there was no difference in the groups for the number of produced words. This result is inconsistent with previous studies that show a positive effect of the combined training also in the production of new words. A possible explanation of this result could be due to the heterogeneity of RTT: Subjects with RTT vary widely in their skill levels. For example, some individuals walk, while others are non-ambulatory. Some have preserved hand function and can participate in feeding or dressing, while others require assistance in all activities of daily living. Some individuals produce words, many use augmentative communication systems of varying complexity, while others have great difficulty in communicating basic needs. Despite this result, our findings related to language are important, as evidence is being gathered on the effects of tDCS plus cognitive empowerment for the production of vowels and phonemes in subjects with RTT, and the results are promising. However, future studies should clarify what the components of best practice are and use them as a guide for the development of combined treatment plans for subjects with RTT.

With reference to the neurophysiological parameters, it was found that the non-sham tDCS group showed an increase in beta and alpha bands, whereas, the sham group presented a tendency to be more random at its functional connections. Probably, the constant electric current, produced by tDCS at 2 mA, induced shifts in the resting threshold of neurons, resulting in secondary changes in cortical activity. This result is in line with recent studies indicating that, in healthy subjects of the non-sham tDCS group, the central EEG electrodes Cz, C3 and C4 turned out to be highly connected within alpha and beta frequency bands. However, to our knowledge this is the first study to show that tDCS can induce positive effects in beta and alpha band of subjects with RTT; for this reason, the underlying cortical mechanism remains poorly understood. Future studies in this direction are needed.

Taken together, the results of this study indicate that combined intervention, tDCS plus cognitive empowerment, is effective in improving attention and language abilities of patients with RTT. A possible explanation for our results is that non-sham tDCS has the potential to modulate brain networks underlying the performance of cognitive and language tasks [42]. The mechanisms underlying the effects of tDCS are not yet understood but may involve changes in the neuromodulation efficacy of different neurotransmitters [43]. The induced excitability changes could persist after the end of the tDCS stimulation, with a duration varying as a function of tDCS parameters [44]. In this work too, a month after tDCS application in the non-sham tDCS group, improvement persisted. A possible hypothesis is that these long-lasting changes can be mediated through NMDA receptor activity [45] and represent a crucial issue of the potential application of this technique in the rehabilitation field. However, now this question is still open in the debate on the factors that affect the neurological mechanism of tDCS.

This study has clinical implications. The evidence from this work suggests that the combined intervention of 10 consecutive weekdays is effective in the improvement of both attention and language abilities in subjects with RTT. This proposes to include tDCS in functional targeting training programs by combining them with tDCS [27]. Future studies, based on larger patient samples and including sham and non-sham tDCS groups, as in the present work, should be conducted to identify the optimal parameters for a useful combined (language or cognitive or motor training plus tDCS) treatment protocol [46].

The present study provides additional support for the use of tDCS as a promising and alternative approach in the RTT rehabilitation field. Our results are encouraging and indicate that combined intervention can help subjects with RTT maximize their capacities. For these reasons, the use of non-sham tDCS with traditional training can be a valid and safe treatment [24]. tDCS is a non-invasive intervention, there is a dearth of safety and effectiveness data in patients with multiple disabilities. Safety is here operationally defined by, and limited to, the absence of evidence of a serious adverse effect [47]. In accordance with previous studies, we did not find side effects, and thus, supporting data on the safety of tDCS [48]. However, caution must be exercised in the interpretation of results, as this study has some limitations. The sample size is small and there may be constraints to the generalizability of the results. However, the effect size is adequate; consequently, the results from groups can be considered reliable and should be validated by a larger sample size. Moreover, in this study, we only considered two parameters, language and attention. Although this work is the first aimed to examine the effects of tDCS combined with cognitive empowerment applied to attention in subjects with RTT, future studies should investigate the potential effects of combined intervention also on other cognitive abilities.

Another note of caution is related to the application criteria of tDCS, for example, the presence of epilepsy. Although the application of tDCS to epilepsy is an emerging non-invasive neuromodulation therapy, there are conflicting results in terms of efficacy and safety [49,50,51,52]. For this reason, we suggest that future research should be undertaken in this area, involving RTT patients with and without epilepsy.

In summary, further larger studies are needed to define appropriate designs and the best stimulation protocols in RTT, and to understand limitations which might hamper scientific rigor and the use of tDCS plus cognitive empowerment as a novel neurorehabilitation strategy also in genetic syndromes, such as RTT.

## Figures and Tables

**Table 1:** Characteristics of two groups.

Group		Name	Clinical Stage	Age	MeCP2 Mutation	Level 1 of Severity
Non-sham	1	M.R.	IV	35	T158M	Moderate
tDCS	2	A.S.	III	13	c.1566_1197 del41	Moderate
	3	S.B.	III	20	c.916C>T	Mild
	4	N.R.	IV	29	R255C	Moderate
	5	T.C.	III	18	R270X	Moderate
	6	G.V.	III	13	R255C	Mild
	7	S.B.	IV	31	R255C	Moderate
	8	S.C.	III	16	T158M	Moderate
	9	R.O.	III	19	R270X	Severe
	10	S.M.	III	18	C.916C>T	Moderate
	11	P.S.	III	15	R255X	Moderate
	12	C.A.	III	19	PT158M	Moderate
	13	T.M.	III	26	R270X	Moderate
	14	V.A.	III	15	R294X	Moderate
	15	E.M.	III	14	R270X	Moderate
	16	E.P.	IV	29	P.R135C	Mild
	17	M.L.	III	25	p.Pro322Leu	Moderate
	18	C.T.	III	17	R270X	Moderate
Sham	19	S.L.	III	18	p.R133C	Mild
tDCS	20	M.G.	III	17	R168X	Mild
	21	V.A.	IV	35	R270X	Mild
	22	M.B.	III	18	C.916C>T	Moderate
	23	A.L.	III	17	T158M	Severe
	24	R.S.	III	21	R168X	Moderate
	25	S.P.	III	16	168RX	Moderate
	26	R.B.	III	18	R270X	Moderate
	27	M.C.	III	19	R168X	Moderate
	28	C.S.	III	13	R306C	Moderate
	29	V.S	III	16	R133C	Moderate
	30	Z.F	III	14	P322A	Moderate
	31	G.S.	III	19	R255C	Moderate

**Table 2:** Means and (Standard Deviation) of attention, production of vowels, phonemes, words, and EEG parameters for the non-sham tDCS and sham tDCS groups.

Parameters	Non-Sham tDCS Group	Sham tDCS Group
	M (S.D.)	M (S.D.)
Attention		
Pre-test	4.00 (2.40)	1.70 (2.07)
Post-test	8.15 (2.66)	2.62 (2.08)
Follow-up	8.00 (2.38)	2.52 (2.12)
Vowels		
Pre-test	0.65 (0.54)	0.46 (1.03)
Post-test	1.92 (1.23)	0.53 (1.50)
Follow-up	1.66 (0.79)	0.53 (1.70)
Phonemes		
Pre-test	1.23 (0.99)	1.15 (0.89)
Post-test	2.60 (1.89)	1.92 (1.85)
Follow-up	2.36 (1.71)	1.80 (1.67)
Words		
Pre-test	0.32 (0.54)	0.15 (0.58)
Post-test	0.80 (1.08)	0.40 (1.05)
Follow-up	1.00 (1.71)	0.35 (1.78)
Alpha (8–13 Hz)		
Pre-test	8.60 (1.75)	8.51 (1.74)
Post-test	9.45 (1.73)	8.51 (1.73)
Follow-up	8.80 (1.72)	8.23 (1.73)
Beta (14–29 Hz)		
Pre-test	14.12 (3.60)	14.02 (3.40)
Post-test	21.43 (3.30)	16.34 (3.30)
Follow-up	19.71 (3.38)	14.05 (3.40)
Theta (3.5–7 Hz)		
Pre-test	6.11 (1.90)	6.01 (1.89)
Post-test	6.22 (1.79)	6.03 (1.80)
Follow-up	6.18 (1.79)	6.05 (1.80)

**Table 3:** Post hoc comparisons for each phase and parameter in the non-sham tDCS and sham tDCS groups.

Parameters	Non-Sham tDCS Group	Sham tDCS Group
	t (p)	t (p)
Attention		
Pre-test vs post-test	16.32 (0.001)	1.90 (0.07)
Pre-test vs follow-up	13.12 (0.001)	1.87 (0.08)
Post-test vs follow-up	2.34 (0.05)	1.75 (0.12)
Vowels		
Pre-test vs post-test	6.27 (0.001)	1.45 (0.03)
Pre-test vs follow-up	5.60 (0.001)	0.85 (0.07)
Post-test vs follow-up	3.01 (0.001)	1.70 (0.08)
Phonemes		
Pre-test vs post-test	6.09 (0.001)	2.25 (0.08)
Pre-test vs follow-up	4.63 (0.001)	2.67 (0.07)
Post-test vs follow-up	1.18 (0.07)	3.56 (0.12)
Words		
Pre-test vs post-test	3.57 (0.01)	2.40 (0.06)
Pre-test vs follow-up	3.16 (0.05)	1.90 (0.11)
Post-test vs follow-up	1.85 (0.05)	2.45 (0.22)
Alpha (8–13 Hz)		
Pre-test vs post-test	14.71 (0.001)	1.12 (0.17)
Pre-test vs follow-up	13.50 (0.001)	2.08 (0.07)
Post-test vs follow-up	12.56 (0.05)	2.70 (0.07)
Beta (14–29 Hz)		
Pre-test vs post-test	6.02 (0.001)	2.14 (0.09)
Pre-test vs follow-up	6.12 (0.001)	1.23 (0.08)
Post-test vs follow-up	1.90 (0.05)	2.18 (0.08)
Theta (3.5–7 Hz)		
Pre-test vs post-test	3.50 (0.09)	1.05 (0.12)
Pre-test vs follow-up	3.55 (0.07)	2.13 (0.08)
Post-test vs follow-up	3.40 (0.07)	1.21 (0.09)

## References

[1] Amir R., Van den Veyver I., Wan M., Tran C.Q., Francke U., Zoghbi H.Y. (1999). Rett syndrome is caused by mutations in X-linked MECP2, encoding methyl-CpG-binding protein 2. Nat. Genet..

[2] Amir R.E., Zoghbi H.Y. (2000). Rett syndrome: Methyl-CpG-binding protein 2 mutations and phenotype–genotype correlations. Am. J. Med. Genet..

[3] Kaufmann W.E., Johnston M.V., Blue M.E. (2005). MECP2 expression and function during brain development: Implications for Rett syndrome’s pathogenesis and clinical evolution. Brain Dev..

[4] Pini G., Bigoni S., Engerström I.W., Calabrese O., Felloni B., Scusa M.F., Di Marco P., Borelli P., Bonuccelli U., Julu P.O. (2012). Variant of Rett Syndrome and CDKL5 Gene: Clinical and Autonomic Description of 10 Cases. Neuropediatrics.

[5] Neul J.L., Kaufmann W.E., Glaze D.G., Christodoulou J., Clarke A.J., Bahi-Buisson N., Leonard H., Bailey M.E.S., Schanen N.C., Zappella M. (2010). Rett syndrome: Revised diagnostic criteria and nomenclature. Ann. Neurol..

[6] Castelli I., Antonietti A., Fabio R.A., Lucchini B., Marchetti A. (2013). Do rett syndrome persons possess theory of mind? Some evidence from not-treated girls. Life Span. Disab..

[7] Fabio R.A., Caprì T., Buzzai C., Pittalà V., Gangemi A. (2019). Auditory and Visual Oddball Paradigm Evaluated Through P300 in Five Girls with Rett Syndrome. Neuroquantology.

[8] Fabio R.A., Giannatiempo S., Oliva P., Murdaca A.M. (2011). The Increase of Attention in Rett Syndrome: A Pre-Test/Post-Test Research Design. J. Dev. Phys. Disab..

[9] Fabio R.A., Giannatiempo S., Caprì T. (2019). Attention and identification of the same and the similar visual stimuli in Rett Syndrome. Life Span. Disabil..

[10] Fabio R.A., Magaudda C., Caprì T., Towey G., Martino G. (2018). Choice Behavior in Rett Syndrome: The consistency parameter. Life Span. Disab..

[11] Hagberg B. (2002). Clinical manifestations and stages of Rett syndrome. Ment. Retard. Dev. Disabil. Res. Rev..

[12] Vignoli A., Fabio R.A., La Briola F., Giannatiempo S., Antonietti A., Maggiolini S., Canevini M.P. (2010). Correlations between neurophysiological, behavioral, and cognitive function in Rett syndrome. Epilepsy Behav..

[13] Pelentsov L.J., Laws T.A., Esterman A.J. (2015). The supportive care needs of parents caring for a child with a rare disease: A scoping review. Disabil. Health J..

[14] Boban S., Wong K., Epstein A., Anderson B., Murphy N., Downs J., Leonard H. (2016). Determinants of sleep disturbances in Rett syndrome: Novel findings in relation to genotype. Am. J. Med. Genet..

[15] Fabio R.A., Colombo B., Russo S., Cogliati F., Masciadri M., Foglia S., Antonietti A., Tavian D. (2014). Recent Insights into Genotype-Phenotype Relationships in Patients with Rett Syndrome Using a Fine Grain Scale. Res. Dev. Disabil..

[16] Fabio R.A., Caprì T., Lotan M., Towey G.E., Martino G., Urbano K.V. (2018). Motor abilities are related to the specific genotype in Rett Syndrome. Advances in Genetics Research.

[17] Fabio R.A., Giannatiempo S., Antonietti A., Budden S. (2009). The role of stereotypies in overselectivity process in Rett syndrome. Res. Dev. Disabil..

[18] Ardolino G., Bossi B., Barbieri S., Priori A. (2005). Non-synaptic mechanisms underlie the after-effects of cathodal transcutaneous direct current stimulation of the human brain. J. Physiol..

[19] Marangolo P., Fiori V., Calpagnano M.A., Campana S., Razzano C., Caltagirone C., Marini A. (2013). tDCS over the left inferior frontal cortex improves speech production in aphasia. Front. Hum. Neurosci..

[20] Gangemi A., Colombo B., Fabio R.A., Berhardt L.V. (2019). The role of Transcranial direct current stimulation in patients with moderate cognitive impairment. Advances in Medicine and Biology.

[21] Kang E.K., Kim Y.K., Sohn H.M., Cohen L.G., Paik N.J. (2011). Improved picture naming in aphasia patients treated with cathodal tDCS to inhibit the right Broca’s homologue area. Restor. Neurol. Neurosci..

[22] Lee S.Y., Cheon H.J., Yoon K.J., Chang W.H., Kim Y.H. (2013). Effects of dual transcranial direct current stimulation for aphasia in chronic stroke patients. Ann. Rehabil. Med..

[23] Shah P.P., Szaflarski J.P., Allendorfer J., Hamilton R.H. (2013). Induction of neuroplasticity and recovery in post-stroke aphasia by non-invasive brain stimulation. Front. Hum. Neurosci..

[24] Nitsche M.A., Liebetanz D., Antal A., Lang N., Tergau F., Paulus W. (2003). Modulation of cortical excitability by weak direct current stimulation--technical, safety and functional aspects. Suppl. Clin. Neurophysiol..

[25] Emara T.H., Moustafa R.R., Elnahas N.M., Elganzoury A.M., Abdo T.A., Mohamed S.A., Eletribi M.A. (2010). Repetitive transcranial magnetic stimulation at 1Hz and 5Hz produces sustained improvement in motor function and disability after ischaemic stroke. Eur. J. Neurol..

[26] Price A.R., McAdams H., Grossman M., Hamilton R.H. (2015). A Meta-analysis of Transcranial Direct Current Stimulation Studies Examining the Reliability of Effects on Language Measures. Brain Stimul..

[27] Roncero C., De Caro M., Thiel A., Probst S., Chertkow H. (2019). Maximizing the Treatment Benefit of tDCS in Neurodegenerative Anomia. Front. Neurosci..

[28] Gangemi A., Caprì T., Fabio R.A., Puggioni P., Falzone A.M., Martino G., Urbano K.V. (2018). Transcranial Direct Current Stimulation (tDCS) and Cognitive Empowerment for the functional recovery of diseases with chronic impairment and genetic etiopathogenesis. Advances in Research.

[29] Fabio R.A., Gangemi A., Caprì T., Budden S., Falzone A. (2018). Neurophysiological and cognitive effects of Transcranial Direct Current Stimulation in three girls with Rett Syndrome with chronic language impairments. Res. Dev. Disabil..

[30] Antal A., Paulus W. (2013). Transcranial alternating current stimulation (tACS). Front. Hum. Neurosci..

[31] Giordano J., Bikson M., Kappenman E.S., Clark V.P., Coslett H.B., Hamblin M.R., Hamilton R., Jankord R., Kozumbo W.J., McKinley R.A. (2017). Mechanisms and Effects of Transcranial Direct Current Stimulation. Dose Response.

[32] Fabio R.A., Castelli I., Marchetti A., Antonietti A. (2013). Training communication abilities in Rett Syndrome through reading and writing. Front. Psychol..

[33] Fabio R.A., Caprì T., Nucita A., Iannizzotto G., Mohammadhasani N. (2018). Eye gaze digital games to improve motivational and attentional ability in Rett syndrome. J. Spec. Educ. Rehab..

[34] Fabio R.A., Caprì T., Martino G. (2019). Understanding Rett Syndrome. Routledge Psychology Taylor and Francis.

[35] Houwen S., van der Putten A., Vlaskamp C. (2014). A systematic review of the effects of motor interventions to improve motor, cognitive, and/or social functioning in people with severe or profound intellectual disabilities. Res. Dev. Disabil..

[36] Romano A., Caprì T., Semino M., Bizzego I., Di Rosa G., Fabio R.A. (2019). Gross Motor, Physical Activity and Musculoskeletal Disorder Evaluation Tools for Rett Syndrome: A Systematic Review. Dev. Neurorehab..

[37] Fabio R.A., Billeci L., Crifaci G., Troise E., Tortorella G., Pioggia G. (2016). Cognitive training modifies frequency EEG bands and neuropsychological measures in Rett syndrome. Res. Dev. Disabil..

[38] Sparrow S.S., Cicchetti D.V., Balla D.A. (2008). Vineland Adaptive Behavior Scales: Second edition (Vineland II), The Expanded Interview Form. Livonia MN: Pearson Assessments.

[39] Fabio R.A., Martinazzoli C., Antonietti A. (2005). Costruzione e standardizzazione dello strumento ‘‘R.A.R.S.” (Rett Assessment Rating Scale). Ciclo Evol. Disabil..

[40] Fanzago F. (1983). Test di valutazione dell’articolazione. Quad Acta Phoniatr Lat..

[41] Welch P.D. (1967). The use of fast Fourier transform for the estimation of power spectra: A method based on time averaging over short, modified periodograms. Ieee Trans. Audio Electroacous.

[42] Nitsche M.A., Cohen L.G., Wasserman E.M., Priori A., Lang N., Antal A., Paulus W., Hummel F., Boggio P.S., Fregni F. (2008). Transcranial direct current stimulation: State of the art 2008. Brain Stimul..

[43] Dayan E., Censor N., Buch E.R. (2013). Noninvasive brain stimulation: From physiology to network dynamics and back. Nat. Neurosci..

[44] Nitsche M.A., Paulus W. (2000). Excitability changes induced in the human motor cortex by weak transcranial direct current stimulation. J. Physiol..

[45] Liebetanz D., Nitsche M.A., Tergau F., Paulus W. (2002). Pharmacological approach to the mechanisms of transcranial DC-stimulation-induced after-effects of human motor cortex excitability. Brain.

[46] Brunoni A.R., Nitsche M.A., Bolognini N., Bikson M., Wagner T., Merabet L., Edwards D.J., Valero-Cabre A., Rotenberg A., Pascual-Leone A. (2012). Clinical research with transcranial direct current stimulation (tDCS): Challenges and future directions. Brain Stimul..

[47] Bikson M., Grossman P., Thomas C., Zannou A.L., Jiang J., Adnan T., Mourdoukoutas A.P., Kronberg G., Truong D., Boggio P. (2016). Safety of Transcranial Direct Current Stimulation: Evidence Based Update 2016. Brain Stimul..

[48] Russo C., Souza Carneiro M.I., Bolognini N., Fregni F. (2017). Safety Review of Transcranial Direct Current Stimulation in Stroke. Neuromodulation.

[49] San-Juan D., Morales-Quezada L., Orozco Garduño A.J., Alonso-Vanegas M., González-Aragón M.F., Espinoza López D.A., Vázquez Gregorio R., Anschel D.J., Fregni F. (2015). Transcranial Direct Current Stimulation in Epilepsy. Brain Stimul..

[50] Vicario C.M., Salehinejad M.A., Felmingham K., Martino G., Nitsche M.A. (2019). A systematic review on the therapeutic effectiveness of non-invasive brain stimulation for the treatment of anxiety disorders. Neurosc. Biobehav. R.

[51] Caprì T., Fabio R.A., Iannizzotto G., Nucita A. (2020). The TCTRS Project: A Holistic Approach for Telerehabilitation in Rett Syndrome. Electronics.

[52] Fabio R.A., Martino G., Caprì T., Giacchero R., Giannatiempo S., La Briola F., Banderali G., Canevini M.P., Vignoli A. (2018). Long chain poly-unsaturated fatty acid supplementation in Rett Syndrome: A randomized placebo-controlled trial. Asia. J. Clin. Nutr..

